# The Use of Finite Element Analyses to Design and Fabricate Three-Dimensional Scaffolds for Skeletal Tissue Engineering

**DOI:** 10.3389/fbioe.2017.00030

**Published:** 2017-05-17

**Authors:** Wim. J. Hendrikson, Clemens. A. van Blitterswijk, Jeroen Rouwkema, Lorenzo Moroni

**Affiliations:** ^1^Department of Tissue Regeneration, MIRA Institute for Biomedical Technology and Technical Medicine, University of Twente, Enschede, Netherlands; ^2^Complex Tissue Regeneration Department, MERLN Institute for Technology Inspired Regenerative Medicine, University of Maastricht, Maastricht, Netherlands; ^3^Department of Biomechanical Engineering, MIRA Institute for Biomedical Technology and Technical Medicine, University of Twente, Enschede, Netherlands

**Keywords:** finite element analysis, additive manufacturing, skeletal regeneration, scaffolds, mechano-regulation

## Abstract

Computational modeling has been increasingly applied to the field of tissue engineering and regenerative medicine. Where in early days computational models were used to better understand the biomechanical requirements of targeted tissues to be regenerated, recently, more and more models are formulated to combine such biomechanical requirements with cell fate predictions to aid in the design of functional three-dimensional scaffolds. In this review, we highlight how computational modeling has been used to understand the mechanisms behind tissue formation and can be used for more rational and biomimetic scaffold-based tissue regeneration strategies. With a particular focus on musculoskeletal tissues, we discuss recent models attempting to predict cell activity in relation to specific mechanical and physical stimuli that can be applied to them through porous three-dimensional scaffolds. In doing so, we review the most common scaffold fabrication methods, with a critical view on those technologies that offer better properties to be more easily combined with computational modeling. Finally, we discuss how modeling, and in particular finite element analysis, can be used to optimize the design of scaffolds for skeletal tissue regeneration.

## Introduction

Mechanical signals play an important role in cell differentiation (Engler et al., 2006), tissue development (O’Reilly and Kelly, 2016), and tissue homeostasis (Horsnell and Baldock, 2016). This is especially the case for skeletal tissue where bone remodeling is governed by the loads that the tissue experiences. In tissue engineering, mechanical stimulation can be used to enhance or control tissue development. However, in order to optimize stimulation regimes, it is important to understand and control how external stimuli, like compression and fluid perfusion, are translated to mechanical signals at the cellular level. After a brief historical perspective on bone mechanobiology and the theories and models that have been formulated over the past two centuries, we discuss scaffold fabrication methods in correlation with the use of finite element analysis (FEA) to design structures that better mimic the native environment. We elaborate on how the combination of developments in FEA to predict and design the local mechanical environment, and additive manufacturing (AM) to fabricate three-dimensional scaffolds with a high level of architectural control, has enabled the use of smart and biomimetic scaffold design for skeletal tissue regeneration.

## Mechanical Stimuli and Tissue Formation

### A Short Historical Perspective

**Figure 1:**
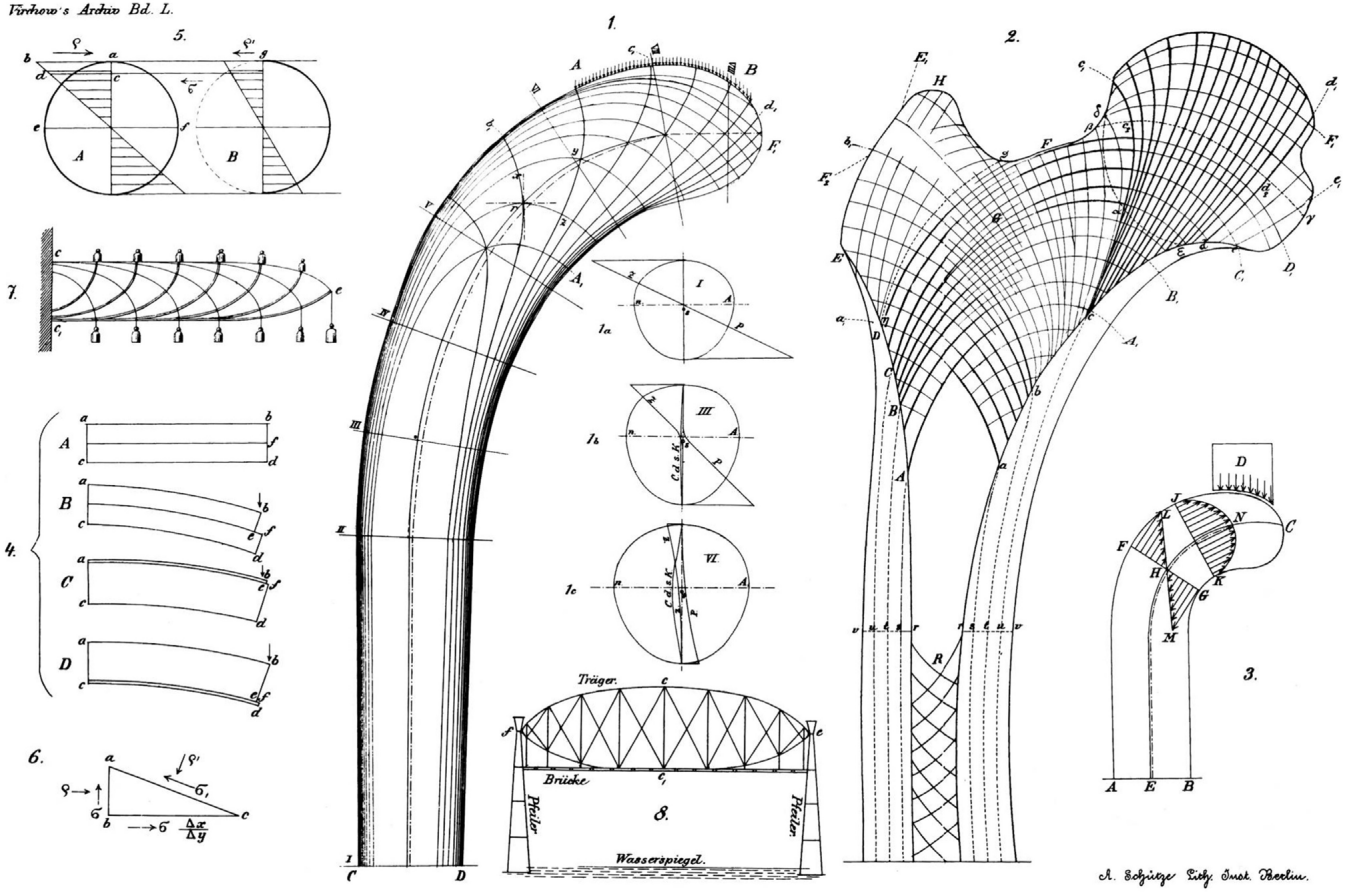
**Stress lines in a crane as calculated and shown by Culmann (left) and the orientation of trabecular bone in a human femur as shown by Meyer (right)**. Reprinted from Skedros and Brand (2011) with permission.

Our current understanding of bone tissue starts with scientific research conducted as early as the 1800s when bone was investigated for its functional adaptation and internal organization. In 1832, Bourgery realized that bone complied with the maximum strength, minimum material principle, which explains the presence of cortical and trabecular bone. In 1866, the engineer Culmann and the anatomist Meyer showed the remarkable resemblance between the direction of the principle stresses in a crane and the bone architecture of the femur (Figure 1). Four years later, Wolff used the observations of Culmann and Meyer to explain the architecture of bone with mechanical stresses. According to Wolff, high stress values correspond with compact bone, less stress with trabecular bone, while no stress means a medullary cavity. In 1881, Roux postulated that bone was self-regulating. It was Wolff in 1892, however, who combined his own observations with those of Bourgery and Roux, leading to the working principles of bone adaptation to external loading, now generally known as Wolff’s law (Roesler, 1987). However, Roux realized 3 years later that functional adaptation was not only restricted to bone but was also applicable to other supportive tissues, such as cartilage and connective tissue. He postulated that the functional mechanical stimulus for bone was compression, while tension resulted in connective tissue, and shearing together with either compression or tension resulted in cartilage (Pauwels, 1960). Although Roux first described a relation between functional stimuli, cell differentiation, and tissue formation, correlating the application of mechanically functional stimuli for specific tissues formation has been a topic of debate since research on the effect of mechanical stimuli on tissue formation was initiated.

**Figure 2:**
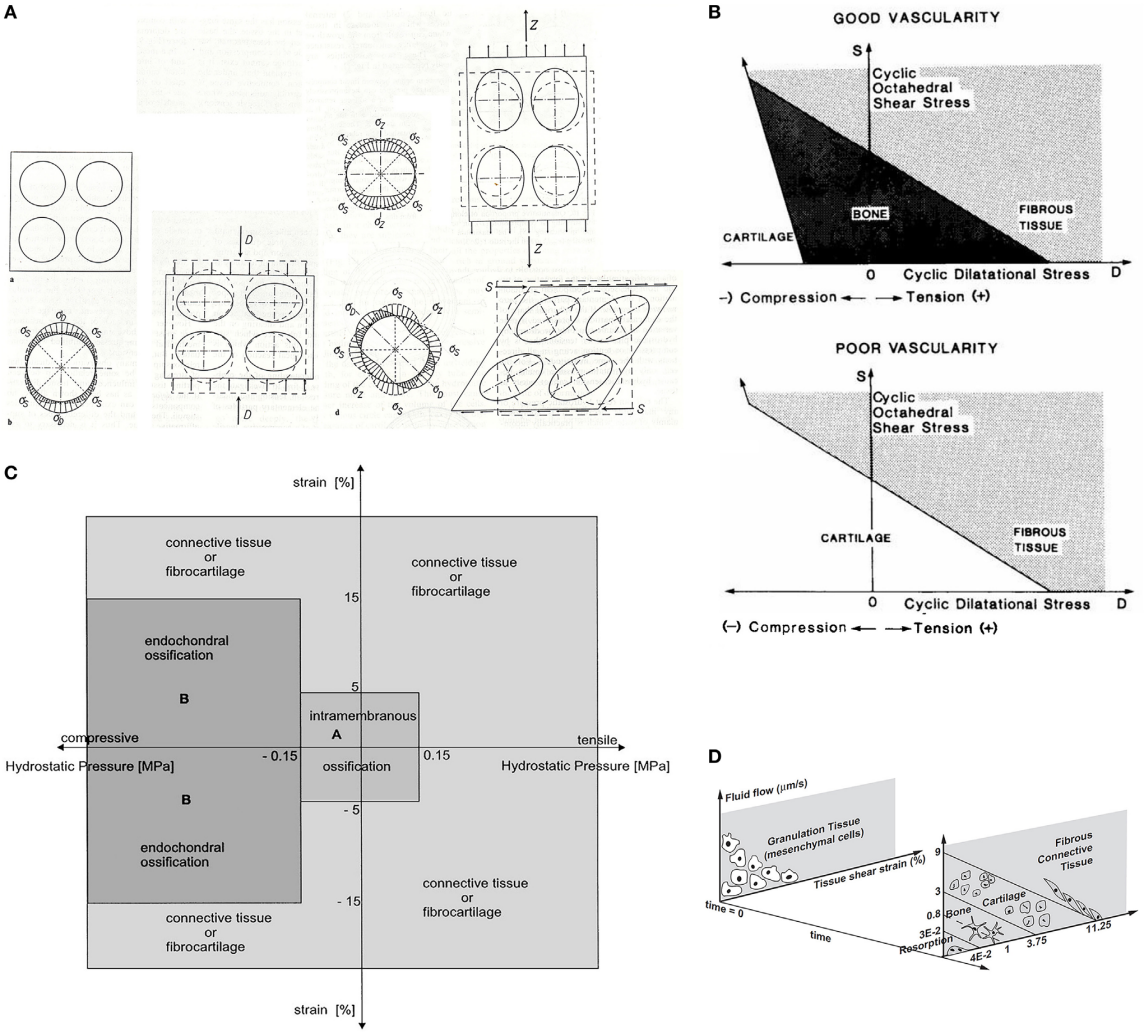
**Multiple mechano-regulation models that predict tissue development based on different mechanical stimuli**. Panel **(A)** shows the model of Pauwels where an elementary sphere suspended in mesenchyme tissue subjected to compression, tension, and shear causes cells to form collagen fibrils. Hydrostatic pressure results in chondrogenesis while bone is formed on an existing solid framework. Panel **(B)** shows the model of Carter. With good vascularization, low distortional (octahedral shear stress), and volumetric (hydrostatic pressure) stresses results in bone formation, intermediate stresses in cartilage, and high stresses in fibrous tissue. Poor vascularization inhibits bone formation. Panel **(C)** shows the model of Claes and Heigele which dictates that low volumetric stress (0.15 MPa hydrostatic pressure) and low local distortional strain (5%) results in intramembranous ossification while hydrostatic pressures <−0.15 MPa with local strains between −15 and 15% results in endochondral ossification. Other hydrostatic pressures and strains results in connective tissue or fibrocartilage. Panel **(D)** shows the model of Prendergast where a distortional strain (tissue or octahedral shear strain) and interstitial fluid flow through mesenchyme tissue stimulates the mesenchymal cell to differentiate and form a tissue. The cell and formed tissue will be replaced by other cell types and tissue based on the perceived biophysical stimuli. Replacement of the cell and tissue population continues until the formed tissue withstands the biophysical stimuli and an equilibrium occurs. Adapted with permission from Pauwels (1960), Carter et al. (1988), Claes and Heigele (1999), and Lacroix and Prendergast (2002), respectively.

More than 60 years later, in 1965, Pauwels postulated a new theory (Figure 2A) based on clinical observations of fracture healing, where cells in the mesenchyme tissue subjected to either pure tension, compression, or shearing elongate. Cell elongation causes the formation of collagen fibrils and, thus, connective tissue (Pauwels, 1960). Hydrostatic pressure on the cell, caused either by swelling of the cell or homogenous compressive forces on the mesenchyme tissue, results in cartilage. In Pauwels theory, bone is not associated to any mechanical stimulus as it was formed on an existing solid framework, either on existing bone, connective tissue, or cartilage.

### The Introduction of FEA

Not until 1981, Hayes and Snyder showed for the first time the highly significant correlation between bone structure and mechanical stresses with FEA. It became increasingly apparent that mechanical loading induced a local signal for cells to respond to. With the emergence of FEA, the research on mechanical loading and cell differentiation increased. Mechano-regulation theories were proposed in which biophysical stimuli were related with cell differentiation and tissue formation (Carter et al., 1988, 1998; Prendergast et al., 1997; Claes and Heigele, 1999). Carter was one of the first to support his theory based on the prediction of fracture healing with FEA (Figure 2B) (Carter et al., 1988). Besides mechanical loading, the entire history of cyclic loading on the mesenchyme tissue of the fracture callus and the vascularity of the mesenchyme tissue were equally important in the formation of tissue. Bone, cartilage, or fibrous tissues were based on two cyclic stresses, the distortional and the volumetric stress.

Claes and Heigele (1999) proposed a quantitative mechano-regulation theory also based on fracture healing (Figure 2C). Their theory distinguished itself from others by the fact that values were given for the mechanical stimuli thresholds for cell differentiation. Also, they proposed that new bone was formed on existing bone or along calcified tissue and that the local strains and stresses dictated the tissue formation. Prendergast modeled tissue formation at the bone–implant interface from a biphasic point of view (Figure 2D) (Prendergast et al., 1997; Lacroix and Prendergast, 2002). The theory assumes that cell populations can be replaced by other cell populations based on the biophysical stimuli perceived. Analyses continued until the formed tissue was able to support the applied biophysical loading. The biophysical stimuli were described by the deformation of the solid part with the octahedral shear strain, and the application of interstitial fluid flow through the mesenchyme tissue.

A study, where tissue differentiation in a torsional loading environment was compared with the predictive models previously developed, showed that the models were not able to predict the tissue differentiation in complete agreement with *in vivo* observations (Isaksson et al., 2006). However, the theory of Prendergast with its octahedral shear strain and interstitial fluid velocity seemed to be the most accurate. A different study where the prediction of tissue differentiation with FEA was compared with *in vivo* bone formation in a bone chamber did also not result in a full validation of the model (Khayyeri et al., 2009). The variability between the *in vivo* samples, which were probably due to genetic variability, were not reflected in the *in silico* predictions. Yet, since its formulation, the theory of Prendergast has been used to develop mechano-biological models to investigate the influence of mechanical loading (Pérez and Prendergast, 2007), angiogenesis (Checa and Prendergast, 2009), cell migration and proliferation (Pérez and Prendergast, 2007; Byrne et al., 2011), and cell sensitivity to mechanical loading (Khayyeri et al., 2011) between different species (Checa et al., 2011) on tissue differentiation to explain the observed differences.

### Mechanical Stimulation at Cellular Level for Tissue Engineering and Regenerative Medicine

It is clear that human bone marrow mesenchymal stromal cells (hMSCs) respond to active mechanical stimulation. When hMSCs were seeded on a 2D silicone sheet, an applied 2–8% uniaxial strain through stretching resulted in osteogenic differentiation (Jagodzinski et al., 2004; Haasper et al., 2008). Another experiment applying uniaxial strain through bending, showed both osteogenic differentiation (Qi et al., 2008) and chondrogenic differentiation (Friedl et al., 2007). Coating of the surface membrane with extracellular matrix (ECM) proteins accelerated osteogenic differentiation with stretching (Huang et al., 2009). With parallel-plate flow chambers, the effect of shear stress due to fluid flow on hMSCs showed to favor osteogenic differentiation as well (Kreke et al., 2008). hMSCs showed to be more sensitive to shear stress after a longer attachment time before fluid shear stress was applied (Yourek et al., 2010).

Numerous studies have also been performed to elucidate the response of cells to mechanical stimulation in a three-dimensional environment. Demineralized bovine bone seeded with hMSCs showed to be susceptive to mechanical loading *in vitro* (Mauney et al., 2004). Osteogenic markers were upregulated and mineralization was increased in the stimulated samples while a further enhancement of osteogenic differentiation was seen when the medium was supplemented with dexamethasone. hMSCs seeded in a three-dimensional collagen matrix and subjected to 10 and 12% tensile strain were driven into osteogenic differentiation without the need of chemical supplements (Sumanasinghe et al., 2006). The non-stimulated controls did not show osteogenic differentiation. In a different study, hydroxyapatite ceramic scaffolds were subjected to dynamic compression of various frequencies (Dumas et al., 2009). Frequencies of 0, 25, 50, or 100 Hz were superimposed on a 3-Hz compression frequency. Compression of 3 Hz alone showed an upregulation of bone specific proteins, which was further amplified with a superimposed 25 Hz frequency. The 50 and 100 Hz reduced osteogenic differentiation showing the responsiveness of cells to compression to be dependent on the strain rate profile as well. A study performed with osteoblast-like cells showed the influence of the frequency of stimulation (Sittichockechaiwut et al., 2009). Short periods of compression were applied to cell seeded poly urethane foam scaffolds on only a few days during the entire culture period of 20 days. Three days of applying a mechanical load was sufficient to induce a faster ECM maturation.

The effect of shear stress on cell differentiation was shown using different experimental set-ups (Kreke et al., 2008). Titanium fiber meshes seeded with hMSCs and subjected to a continuous fluid perfusion in parallel-plate flow chambers, showed osteogenic differentiation (Holtorf et al., 2005). When the medium was supplemented with dexamethasone, the osteogenic differentiation was further enhanced. Synthetic foam scaffolds seeded with hMSCs and suspended in a spinner flask containing dexamethasone supplemented medium had upregulated osteogenic gene expression compared to static cultured scaffolds (Stiehler et al., 2009). Collagen scaffolds seeded with hMSCs were stimulated in either a perfusion bioreactor or spinner flask and showed osteogenic differentiation in both cases (Meinel et al., 2004). The scaffold architecture and fluid flow direction influenced cell differentiation and tissue formation. Additionally, calcium deposits were found in the inner part of the scaffold and showed different deposition orientations based on the fluid flow.

The combination of continuous fluid perfusion and cyclic compression was shown to affect osteogenic differentiation of hMSCs in a decellularized bovine matrix (Jagodzinski et al., 2008). Perfusion alone resulted in a proliferative behavior of the seeded hMSCs, while in combination with mechanical loading hMSCs differentiated toward the osteogenic lineage. Similar results were shown in a study with a collagen scaffold for a meniscus implant. Continuous fluid perfusion in combination with mechanical stimulation improved the biomechanical properties of the *in vitro* tissue-engineered construct (Petri et al., 2012). A porous poly urethane scaffold intended for a meniscus implant also showed the beneficial effect of fluid perfusion and mechanical stimulation on the biomechanical properties of the tissue-engineered construct (Liu et al., 2012). The continuous stimulation resulted in increased ECM deposition and, therefore, higher equilibrium modulus. However, constructs with a lower stimulation time displayed a lower equilibrium modulus than the constructs with a longer stimulation time. A PGA-mesh scaffold subjected to mechanical shear and a simultaneous static compression resulted in improved biochemical properties of the tissue-engineered construct (Shahin and Doran, 2012). After stimulation, GAG and collagen type II synthesis were enhanced when compared to perfusion alone and static cultures.

## Scaffold Fabrication

The role of the scaffold architecture is crucial in order to understand cell differentiation and tissue formation inside skeletal tissue engineering scaffolds. Several fabrication techniques exist for scaffolds used in tissue engineering and regenerative medicine. These included conventional, electrospinning, and AM techniques. Each fabrication technique is linked to specific scaffold properties that affect how the cells within the scaffold respond to external mechanical stimuli. This will be discussed in more detail in the following section.

### Conventional Scaffold Fabrication Techniques

Conventional techniques are based on fabrication technologies in which the biomaterial is mixed with a porogen agent. Some examples are particulate leaching, thermally induced phase separation (TIPS), gas-foaming, and emulsion freeze-drying (Janik and Marzec, 2015). Depending on the technology used, some control over pore size, shape, and interconnectivity is possible by adapting the processing parameters. Especially, TIPS results in highly interconnected tubular pores (Ma and Zhang, 2001). However, since pore formation in conventional scaffold fabrication techniques depends on random nucleation events or random packing of particles, the exact porosity of resulting scaffolds is hard to predict and control. As such, these scaffolds are less fit for applications where an exact scaffold geometry is needed to control the local mechanical signals that cells experience upon scaffold compression or perfusion.

### Electrospinning

Conventional scaffold fabrication techniques lack the possibility of mimicking the structural composition of bone ECM, thus limiting the implementation of the input that can be originated by FEA. With electrospinning, small diameter fibers can be produced in the micro- and nanoscale range mimicking the physical dimensions of natural fibrillar ECM (Boffito et al., 2014; Shabafrooz et al., 2014). An electrical field between the metallic nozzle and the metallic collector created by a high voltage potential ejects a thin liquid polymer solution to the collector. During the travel to the collector, the solvent of the polymer is evaporated and a dry fiber is left on the collector. Fiber properties are dependent on the applied voltage (Deitzel et al., 2001; Meechaisue et al., 2006), flow rate (Megelski et al., 2002), collector distance (Bhardwaj and Kundu, 2010), solution parameters (Henriques et al., 2009), and ambient parameters (Kumbar et al., 2008). The voltage is able to influence the fiber diameter from microns to few tens of nanometers (Deitzel et al., 2001). The flow rate influences the fiber diameter as well, along with the fiber morphology (Megelski et al., 2002). Apart from tuning the diameter of the individual fibers, process parameters can also be adapted in order to create micro- or nanoscale surface features on the fibers (Li and Xia, 2004). By controlling the evaporation of the solvent during electrospinning, a phase separation process can be initiated resulting in surface features, such as grooves (Liu et al., 2014) or nanoporosity (Wang et al., 2013).

### Additive Manufacturing

Although electrospun scaffolds can mimic more closely the physical dimensions of native ECM, scaffolds obtained through electrospinning and conventional techniques have the common problem that the pore network configuration is hard to control. To circumvent this problem, scaffolds can also be fabricated using AM, which is defined as the process of joining materials to make objects from three-dimensional model data, usually in a layer upon layer method (Mota et al., 2015). Such a layer-by-layer method enables to construct scaffolds through the Computer-Aided Design/Computer-Aided Manufacturing (CAD/CAM) principles. This means that pore size, shape, and porosity can be designed to match the needs of a specific application. Among AM platforms are powder-, photosensitive-, and melt-extrusion-based techniques. With powder-based techniques, a powder is bound through, for example, melting [Selective Laser Sintering (SLS)] or a binding material (Printing). In SLS, powder particles of either polymer, ceramic or a composite, are bound through a high-intensity laser beam, after which new powder particles are applied for the next layer (Ciardelli et al., 2005; Williams et al., 2005; Eosoly et al., 2010; Shanjani et al., 2010). The resolution of this technique depends both on the powder and laser beam used. Printing is based on the same technique as SLS, except that the binding occurs through a binding material, such as an organic solvent, that is printed on the powder bed (Butscher et al., 2011). Here, the droplet size determines the minimal possible scaffold fiber diameter and spacing. Through sintering after scaffold fabrication, the mechanical properties can be improved (Shanjani et al., 2010).

Stereolithography (STL) is a photosensitive-based technique in which a light source polymerizes a liquid resin. Similar to printing, the beam traces the contours of the designed scaffold. Upon finishing a layer, the station with the cured resin is lowered and a new layer of liquid resin is placed on the fabricated layer (Hutmacher et al., 2004). Pore size, shape, and porosity can be designed through the CAD/CAM principle and mechanical properties can, therefore, be tuned accordingly (Melchels et al., 2011). However, besides the limitation of available biocompatible photosensitive biomaterials, fabricated scaffolds through STL needs post-processing for a better polymerization and removing unreacted resin (Melchels et al., 2010).

Fused deposition modeling (FDM), three-dimensional fiber deposition (3DF), precision extrusion deposition (PED), and bioscaffolding are examples of extrusion-based AM techniques (Mota et al., 2015). In FDM, a filament of a thermoplastic polymer is shortly heated in the nozzle and extruded on a stationary platform. The main advantage of this technique is the accurate control of the pore configuration of the scaffold. However, only thermoplastic polymers in a filament shape can be used as the base biomaterial, which limits in part the palette of biomaterials that can be processed (Hutmacher, 2000). 3DF is very similar to FDM. However, the polymer is inserted in a cartridge which is heated and subsequently pressurized for extruding the polymer melt onto the platform in a layer-by-layer fashion. The limitation of a thermoplastic polymer in a filament base shape is removed and thermoplastic polymers in any base shape could be used, thus expanding the gamma of biomaterials that can be used (Moroni et al., 2005). The disadvantage of this system is the possible thermal degradation of the polymer due to high residency time in the heated cartridge. Similar to FDM and 3DF, PED uses a screw to extrude the polymer melt on the platform (Shor et al., 2009). The combination of pressurized extrusion (3DF) and screw extrusion (PED) can be found in a commercial AM machine, the Bioscaffolder. This machine has been used to obtain scaffolds with the same pore configuration but from different biomaterials (Chaudhuri et al., 2015; Hendrikson et al., 2015).

### Considerations for FEA

From an FEA perspective, scaffolds should be highly reproducible and the pore configuration highly controllable. Scaffolds obtained through conventional fabrication techniques lack the possibility of complete control of the pore configuration and, therefore, also suffer from limited reproducibility in terms of producing the same identical pore network. Although electrospinning is a very interesting technique, the fiber dimensions, the control of pore configuration, and reproducibility renders electrospun scaffolds not desirable for investigating scaffold properties with FEA. Melt-electrospinning is a rising technology that has attracted a lot of attention for scaffold fabrication as it holds the potential to combine the high fidelity of AM platforms with the ECM-mimicking properties of electrospinning (Gernot et al., 2015). In this respect, it will be very interesting to combine this technology with the more rationale design of scaffolds for bone or other tissue regeneration that can be obtained through FEA. Printing is an interesting option as the pore configuration can be controlled. However, it remains challenging to obtain reproducible scaffolds when the surface topography of the fibers is concerned. Photosensitive techniques are very interesting as the reproducibility and pore configuration will be highly controllable. Despite good combination of computational modeling and design of scaffolds pore network and STL has been achieved (Melchels et al., 2010), the limited availability of biomaterials makes them a less suitable option than extrusion-based techniques. With these techniques the pore configuration is highly controllable and scaffolds are highly reproducible. Additionally, the choice of suitable biomaterials is broader than other AM techniques. Therefore, extrusion-based AM technologies seem the best scaffold fabrication platform to be combined with FEA design.

## Mechanical Requirements for Skeletal Tissue Engineering Scaffolds

### Bone

Scaffolds developed with the intention to serve as bone grafts are generally characterized for their osteogenic potential and not for their mechanical properties. However, there is a consensus that the mechanical properties of the scaffold should match the mechanical properties of bone when the scaffold will be used in a load-bearing application (Fröhlich et al., 2008; Velasco et al., 2015). Therefore, finite element models (FEMs) have been developed to capture the anisotropic mechanical behavior of trabecular bone. By tuning the porosity of the FEMs to match the hierarchical structure of trabecular bone, anisotropic mechanical behavior was predicted similar to trabecular bone (Huang et al., 2014). A different method was applied to develop a scaffold architecture matching the desired porosity and elastic properties of trabecular bone using an optimization algorithm. Scaffolds were fabricated by printing (Lin et al., 2004), casting (Hollister et al., 2002), or selective laser melting (Challis et al., 2010). However, no mechanical tests were performed on the fabricated scaffolds. More recently, a numerical optimization strategy was developed in which the mechanical properties of the scaffold could match native tissue. The novelty of this approach was that the material behavior of degradation and loss of mechanical stiffness were taken into account and used to develop a scaffold with struts of different biomaterials (Heljak et al., 2012).

### Cartilage

The hierarchical structure of cartilage is depth dependent and is usually divided into three zones, the superficial, middle, and deep zone, in which the composition and collagen orientation delineates the zones (Pearle et al., 2005). In the superficial zone, collagen is oriented tangential, while in the deep zone it is oriented perpendicular to the articular surface and the middle zone delineates the transition in the orientation. The composition and orientation of the constituents, gives articular cartilage, and subchondral bone, a range of complex mechanical characteristics and behaviors. The orientation of collagen results in anisotropic mechanical behavior of cartilage (Treppo et al., 2000). Furthermore, collagen can resist tensile loads better than compressive loads.

The anisotropic composition and the visco-elastic behavior of cartilage results in a range of reported mechanical properties. For a human knee, a dynamic stiffness of 4.5 MPa was reported at a loading frequency of 0.1 Hz (Treppo et al., 2000), an aggregate static modulus of 0.1–2.0 MPa (Moutos et al., 2010), while a Young’s modulus of 10 MPa has been used in FEA simulations for cartilage (Lacroix et al., 2002). Electrospun scaffolds have been used to regenerate articular cartilage and meniscus, as the nanofibers supposedly mimic the collagen orientation in articular cartilage (Baker and Mauck, 2007; Wise et al., 2009; Accardi et al., 2013). It was shown that fiber orientation not only influences cell orientation (Wise et al., 2009) but also the mechanical properties of the scaffold (Baker and Mauck, 2007; Accardi et al., 2013). More recently, hydrogels have been fiber reinforced to mimic the articular cartilage even more, obtaining encouraging results compared with equine (Visser et al., 2015) and human articular cartilage (Moutos et al., 2010; Liao et al., 2013).

Swelling of cartilage due to the presence of proteoglycans is counterbalanced by the solid collagenous matrix. The presence of water gives cartilage visco-elastic properties and provides fluid load support. Upon loading of cartilage, fluid in the cartilage is pressurized and supports the load. However, in time, the fluid is expelled from the tissue and the solid matrix supports the load. Besides, this load sharing between the solid and fluid matrix proved to be important for the frictional response of articular cartilage (Caligaris and Ateshian, 2008; Ateshian, 2009). The sliding velocity, contact area, and lubrication are all influential on the friction coefficient (Caligaris and Ateshian, 2008; Gleghorn and Bonassar, 2008; Neu et al., 2008; Ateshian, 2009; Bonnevie et al., 2011; Shi et al., 2011). Combinations of contact pressure, speed, and viscosity (Hersey number) results in different lubrication modes which can be related to the friction coefficient by the Stribeck curve (Gleghorn and Bonassar, 2008). Therefore, the experimental setup has significant influence if such a fluid support is achieved and the resulting friction coefficient measured (Shi et al., 2011). Friction coefficients for bovine articular cartilage were reported between 0.02 and 0.1 (Caligaris and Ateshian, 2008; Bonnevie et al., 2011), while for human cartilage values between 0.06 and 0.15 were reported for a variety of experimental settings (Merkher et al., 2006). Non-cellular woven PCL scaffolds infused with either an alginate or poly acrylamide hydrogel showed to have friction values similar to the higher frictional coefficients of human cartilage (Liao et al., 2013). The same woven PCL scaffolds but now cultured with hMSCs and fibrin showed similar friction coefficients as the non-cellular scaffolds (Moutos and Guilak, 2010). Electrospun scaffolds subjected to physiological loads revealed that fiber orientation in the direction of the loading had lower surface damage compared to perpendicular oriented fibers. *In vitro* cell culture, however, did not reduce the friction coefficient in these scaffolds which were similar to the friction coefficient of a bovine knee joint explant (Accardi et al., 2013).

### Osteochondral Tissue

For osteochondral replacements, scaffold designs are classified upon their structural design. In order to succeed in regenerating osteochondral tissue, a part of the scaffold has to be dedicated to cartilage regeneration and the other part to bone regeneration. Therefore, a scaffold for osteochondral constructs can be classified as either mono-, bi-, or multiphasic from either the scaffold architecture, biomaterial, or composition. In a monophasic scaffold design, there is only one scaffold design for the whole construct and no distinction is made between the cartilage and bone region. A biphasic scaffold can have for example two architectures, two biomaterials, or two porosities, specifically for bone and cartilage. A multiphasic scaffold has more than two different architectures, biomaterials, or porosities in order to obtain an osteochondral construct. The easiest method of obtaining a biphasic scaffold is to combine a scaffold for cartilage regeneration, usually a hydrogel, with a scaffold for bone regeneration, usually a polymer or a ceramic (Gao et al., 2001). However, separation of the constructs *in vivo* is a point of concern (Schek et al., 2004). Ideally, the bond between the different scaffolds should be mimicking the tidemark region between articular cartilage and subchondral bone as seen in native tissue. This implies the need of a multiphasic scaffold in order to provide a stable construct (Jiang et al., 2010). A hydrogel region for cartilage regeneration could be coupled with PLGA microspheres to a region of composite PLGA microspheres for bone regeneration. Mechanical characterization of the construct, however, showed lower values for the construct compared to native tissue (Jiang et al., 2010). Another method of obtaining multiphasic scaffold design is through scaffolds with distinct gradients or porosities for cartilage and bone regeneration. Microspheres loaded with growth factors specific for cartilage or bone regeneration were used to obtain a scaffold with a functional growth factor gradient. Histological analysis showed good results although no mechanical analyses have been performed (Mohan et al., 2011). Combining microspheres with a porogen, a gradient in porosity was achieved for the scaffold (Dorcemus and Nukavarapu, 2014). Alternatively, microspheres with different diameters were used to obtain a scaffold with a gradient in pore size (Tang et al., 2012). While there was a difference in proliferation rate between the different pore sizes, osteogenic differentiation was not statistically different. These studies, therefore, show how challenging it is still to design a functional scaffold for osteochondral tissue regeneration where several physical, chemical, mechanical, and biological different features have to be taken into account. It is also for these more complex tissue engineering and regenerative medicine strategies where multiple tissues are targeted that FEA can be of great help in optimizing the scaffolds design.

## Optimizing Scaffold Design with FEA

Additive manufacturing provides a tool in developing scaffolds with tailored design that can help better matching musculoskeletal tissue regeneration requirements. Pore configuration can be completely controlled and, therefore, tuned to mechanically match native tissue (Moroni et al., 2006a,b). A database has been developed with different structural configurations, in which the combination of configuration and porosity can be used to obtain scaffolds with desirable mechanical properties through SLS (Sudarmadji et al., 2011). Besides the structural configuration to match the mechanical properties with native tissue, cells in the scaffold should be able to migrate throughout the whole scaffold with a sufficient nutrient and waste exchange provided for them to from tissue (Hollister et al., 2002; Shipley et al., 2009). To satisfy these requirements, the porosity and interconnectivity of the pores play an important role (Sengers et al., 2007). Additionally, the pore shape alongside the porosity and interconnectivity play an important role in the stress and strain distribution when mechanically loaded (Dias et al., 2014). Mathematical models have been developed to optimize the scaffolds internal architecture for trabecular bone to obtain the desired mechanical properties and porosity (Hollister et al., 2002; Lin et al., 2004), and fabricated scaffolds displayed a reasonable match with the computational design (Dias et al., 2014).

Using FEMs, the mechanical behavior of a representative pore of a regularly structured titanium scaffold was reproduced (Ryan et al., 2009). FEMs based on μCT scans proved to be a good indicator for the apparent stiffness, but the CAD-based FEMs were not able to capture the experimental results. Similar for the permeability of AM scaffolds, CAD-based FEMs could not predict the permeability of the scaffold accurately compared to the experimental values, while the μCT-based FEMs did (Truscello et al., 2012).

Despite the lack of accurately reproducing experimental results, CAD-based models were used to investigate the influence of scaffold designs on cell differentiation and tissue formation. For this, coupling of the mechano-regulation of Prendergast (Prendergast et al., 1997) with the stress and strain distributions in the scaffold was applied. By using a representative pore of a (theoretical) scaffold as unit-cells, the influence of biomaterial stiffness, porosity, and pore size was determined (Sanz-Herrera et al., 2009). A faster bone formation was seen in a scaffold with a stiffer biomaterial, increased pore sizes, or porosity with the limit of early collapsing with a too large pore size or porosity. Similar behavior was seen in an idealized scaffold whereas also the dissolution rate showed to be important as a too fast dissolution rate collapsed the scaffold (Byrne et al., 2011).

A μCT-scanned calcium phosphate scaffold was used to calculate the stress and strain distribution throughout the scaffold with FEA (Sandino et al., 2008). It not only showed the influence of pore morphology on the stress and strain distribution but also on the stress and strain magnitudes. The heterogeneous pore configuration of the scaffold resulted in areas with high stresses and strains. A later study investigated the effect of loading on cell differentiation and tissue formation in the same μCT-scanned scaffold (Sandino and Lacroix, 2011), showing that the current stimulation values used in *in vitro* studies were higher than necessary. A FEM of a salt-leached scaffold highlighted the effect of irregular pore size and distribution by predicting a heterogeneous stress and strain distribution and subsequently a heterogeneous tissue formation (Milan et al., 2009, 2010). CAD-based FEMs were developed to investigate the influence of pore shape, size, and porosity with various biophysical loadings (Olivares et al., 2009). Similar to the irregular structured FEMs, these FEMs showed the critical influence on the stress and strain distribution and the cell differentiation stimulus. Fluid flow simulations evidenced the influence of the pore shape on shear stress in which small pore sizes resulted in high shear stresses. FEMs based on μCT-scanned AM scaffolds with different pore configurations also showed the influence of pore configuration on the stress and strain distribution and cell differentiation prediction (Hendrikson et al., 2016). Pore size and shape influenced fluid flow and shear stress distribution, while the presence of a supporting column influenced the strain distribution from mechanical compression. For cell differentiation prediction, the fluid flow and mechanical loading each had a distinctive influence based on the pore configuration. Large pore sizes had less influence for fluid flow, while with smaller pore sizes the fluid flow had a stronger influence.

## Current Developments and Future Perspectives

**Figure 3:**
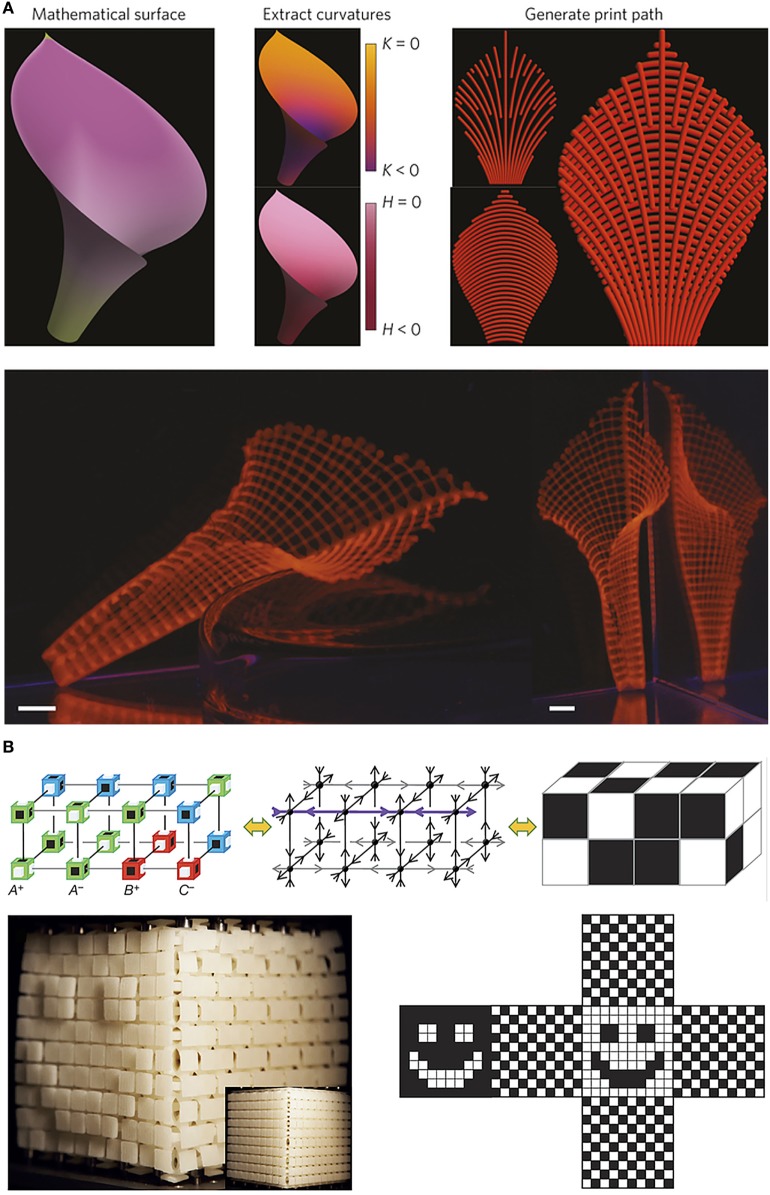
**Examples of current developments where computational modeling is combined with additive manufacturing to acquire advanced functionalities**. Panel **(A)** shows an example of biomimetic 4D printing, where composite hydrogel architectures that are encoded with localized, anisotropic swelling behavior, are printed in designs that result in a predictable and controllable shape change when the objects are hydrated. Panel **(B)** shows an example where anisotropically deforming cubic building blocks are combined using three-dimensional printing. By computationally designing the relative placement of multiple building blocks, predictable surface textures can be created when exposed to uniaxial compression or extension. Adapted with permission from Gladman et al. (2016) and Coulais et al. (2016), respectively.

The field of AM is developing fast. Resolution, as well as the amount of compatible biomaterials, is increasing. This allows for an ever increasing control over the geometry and surface structure, as well as the mechanical properties and the surface chemistry of scaffolds that are produced using AM. Combined with an increasing complexity and computational power of FEMs, and a better understanding of how mechanical signals result in specific cellular behavior and differentiation, this will allow for the design and manufacturing of scaffolds that result in predictable and optimal tissue development upon active mechanical stimulation and fluid perfusion. Apart from that, recent developments regarding the AM of scaffolds using shape memory or conductive materials, results in a further increase of the control over cellular differentiation (Figure 3A) (Gladman et al., 2016). A particular appealing option would be to develop reconfigurable three-dimensional systems able to change structural properties depending on the applied deformation (Figure 3B) (Coulais et al., 2016). On the modeling side, further improvement of current models developed to predict cell activity could combine cellular with genetic information (Bolander et al., 2016). Furthermore, current models focus on a specific tissue formation, without taking into account associated tissues like vascular and neural networks. Whereas, more recent models have also started to look into vasculature predictions (Carlier et al., 2015), innervation remains a field poorly studied in tissue engineering and regenerative medicine.

## Conclusion

With the fast development of modern technologies, CAD/CAM approaches are very realistic methods with which scaffolds can be prepared that possess properties at several multidimensional scales and are usable in tissue engineering and regenerative medicine. Smart use of the combination of computational power and scaffold design can provide valuable and deep insights in the process of tissue formation. FEA could be used to design *a priori* scaffolds with optimal structural, mechanical, and physical properties to direct specific cell function. FEAs could also be used to predict the best culture conditions, in terms of nutrient perfusion and active mechanical stimulation, to further influence cell differentiation. Such combination has the potential to accelerate research in addressing the ultimate goal of tissue engineering: the replacing, engineering, or regenerating human cells, tissues, or organs to restore or establish normal function. Furthermore, the resulting improved engineered constructs would also find broad use in studying skeletal biology in more reliable three-dimensional *in vitro* models, thus holding the potential to offer a replacement to animal studies both for fundamental studies to better understand pathological mechanisms behind skeletal diseases as well as for applied research to find more efficient advanced therapies.

## Author Contributions

WH, JR, and LM structured the literature to be reviewed. WH, JR, CB, and LM wrote the manuscript. WH selected figures and asked for reprint permission.

## Conflict of Interest Statement

The authors declare that the research was conducted in the absence of any commercial or financial relationships that could be construed as a potential conflict of interest.
